# Prevalence of Musculoskeletal Symptoms among Dental Health Workers, Southern Thailand

**DOI:** 10.1155/2016/5494821

**Published:** 2016-08-14

**Authors:** Somsiri Decharat, Piriyalux Phethuayluk, Supandee Maneelok

**Affiliations:** ^1^Department of Industrial Hygiene and Health Science, Faculty of Health and Sports Science, Thaksin University, 222 Moo 2 Papayom District, Phatthalung Province 93210, Thailand; ^2^Department of Public Health, Faculty of Health and Sports Science, Thaksin University, 222 Moo 2 Papayom District, Phatthalung Province 93210, Thailand

## Abstract

*Objectives*. The objective of this study was to describe the socioeconomic situation of dental health work and work characteristics and to evaluate the prevalence of musculoskeletal symptoms among dental health workers.* Material and Methods*. A cross-sectional study was conducted with 124 dental health workers and 124 persons in the reference group, matched to dental health workers by gender, were recruited from the workers who worked at the same 17 community hospitals in Nakhon Si Thammarat province, Thailand. Information was collected by using questionnaire. Data analysis comprised descriptive and analytical components.* Results and Discussion*. 75.8% were female and 24.2% were male dental health workers. 91.9% of subjects had worked >5 years. Most subjects worked for >8 hours per day and worked >6 days per week, at 63.7% and 53.2%, respectively. 100% of subjects worked in public institutions, and 68% also worked in both public and private institutions. Most subjects (52.4%) did not exercise. Daily activity, gender, duration of work, hours worked per day, days worked per week, and physical activity were significantly associated with musculoskeletal symptoms at <0.001.* Conclusion*. The prevention and reduction of MSDs among dentists should include improving their education in dental ergonomics.

## 1. Introduction

Dentistry is recognized to be a demanding occupation. In Thailand, the numbers of dentist increased from 16,137 dentists in 2010 to more than 20,000 in 2014 [1]. In addition, health effects data of the dentists in Thailand was weak. While many studies reported the work related musculoskeletal disorders (WMSDs) in the dental personnel across different countries [2–5], the phenomenon knowledge of dentists in Thailand is lacking. Musculoskeletal symptom (MSS) may be defined as pain commonly experienced by dentists in the course of their career [6]. Studies show that the circumstance of musculoskeletal symptoms among dentists varies. Biller, 1994 [7], presented that 65% of dentists complained of back pain in his study. Similarly, Finsen et al. 1998 [8] reported 65% MSS among Danish dentists and 78% of dentists in Thailand [9]. Part-time Thai dentists were found to have a higher proportion of musculoskeletal problems than their full-time counterparts. The number of years since graduation was also significantly associated with musculoskeletal pain in these Thai dentists. Of 264 dental personnel, the report of MSD pain impacting daily activity was 76.1%. Nithithamthada and Puntumetakul, 2013 [10], reported work related MSDs as 71.2%. The objectives in this study were to describe the socioeconomic situation of dental health work and work characteristics and to evaluate the prevalence of musculoskeletal symptoms among dental health workers.

## 2. Material and Methods

The investigation was a cross-sectional descriptive study. This study was a descriptive comparative survey of two groups of healthcare workers, during May and September 2013.

### 2.1. Study Population and Samples

The study population comprised a subject group and a reference group. The subjects were obtained by a purposive sampling, all of 17 community hospitals in Nakhon Si Thammarat province in South of Thailand. The samples size in this study was calculated by estimating from the total number of dental health workers cases. Thus, the subjects enrolled were 124 dental health workers (30 males and 94 females including 16 dentists, 70 dental hygienists, and 38 dental assistants) who worked at 17 community hospitals in Nakhon Si Thammarat province in South of Thailand. 124 reference groups (e.g., 35 general officers, 25 medical record officers, 37 office cleaners, and 27 security officers), matched to dental health workers by gender, were recruited from the workers who worked at the same 17 community hospitals in Nakhon Si Thammarat province in South of Thailand.

The inclusion criteria of the subject group were dental health workers aged between 20 and 60 years who had at least one-year experience. They agreed to participate in the study and provided written informed consent. This study was approved by the Ethics Committee of the Institute of Research and Development, Thaksin University. Permission to conduct the study was obtained from the community hospitals owners. All of participants received a clear explanation of the purpose of this study and agreed to participate using signed consent forms.

### 2.2. Sample Collection

The questionnaire collected information on the following variables: general information, work characteristics, occupational life style (e.g., duration of work, hours worked per day, and days worked per week), and presence or absence of musculoskeletal symptoms in the previous 12 months (dependent variables). In addition, sociodemographic variables were age, weight, height, and gender. Behavioral variables were prophylaxis and physical activity. Interviews were conducted after work by trained interviewers. Data was confirmed by using direct observation.

### 2.3. Statistical Analysis

Data analysis comprised descriptive and analytical components. In the descriptive component, workers' personal and occupational characteristics are defined and their musculoskeletal symptoms described. The analytical component assessed the relationships between musculoskeletal symptom prevalence and the independent variables described above. All independent and dependent variables were categorical, so chi-square tests were used to assess these relationships. Data analysis was conducted using SPSS for Windows.

## 3. Results

From 17 purposively selected hospitals, 124 subjects and 124 references were interviewed. The mean age of the dental health workers was 37.3 years and the mean age of the reference group was 39.4 years. Duration of work by dental health workers was 15.5 years and 16.8 years for the reference group. Hours worked per day and days worked per week by the dental health workers were 12.3 hours and 6.3 days, respectively. All of the subjects worked in public institutions, and 68% also worked in a private capacity.

Of all respondents in the research study, 75.8% were female and 24.2% were male dental health workers. 91.9% of subjects had worked >5 years. Most subjects worked for >8 hours per day and worked >6 days per week, at 63.7% and 53.2%, respectively. 100% of subjects worked in public institutions, and 68% also worked in both public and private institutions. Most subjects (52.4%) did not exercise.

The proportion of musculoskeletal symptoms that interfered with a dental health worker's daily activities is given in Table 1. The 3 most common symptoms that had interfered with a dental health worker's daily activities during the previous 12 months were at the shoulders (27.4%), the neck (23.4%), and the lower back (22.9%), respectively. The neck pain, the lower back, the shoulders, the hands/wrists, and the knees that interfered with daily activity were significantly more likely to be reported by dental health workers (*P* < 0.001, *P* < 0.001, *P* < 0.001, *P* < 0.001, and *P* < 0.001, resp.). The occurrence of symptoms in the shoulders, the neck, and the lower back was statistically significantly higher among dental health workers than the reference group. The occurrence of symptoms in the shoulders (90.0%), the neck (83.3%), the lower back (50.0%), and the knees (16.7%) was statistically significantly higher among male dental health workers than female dental health workers (*P* < 0.001, *P* < 0.001, *P* < 0.001, and 0.023, resp.). The working position was significantly associated with a higher prevalence of the shoulders, the neck, the lower back, and the knees symptoms as is shown in Table 2.

The occurrence of symptoms in the shoulders (50.%), the neck (41.2%), the lower back (33.3%), the knees (25.4%), and the ankles/feet (9.7%) was statistically significantly higher among dental health workers who worked >5 years compared to dental health workers who worked ≤5 years (*P* < 0.001, *P* < 0.001, *P* < 0.001, *P* < 0.001, and *P* < 0.001, resp.). Thus, the duration of work as >5 years was significantly associated with a higher occurrence of symptoms in the shoulders, neck, lower back, knees, and the ankles/feet. The occurrence of symptoms in the shoulders (67.1%), the neck (59.5%), the lower back (43.0%), the upper back (30.4%), the knees (34.5%), the hands/wrists (27.9%), the elbows (19.0%), and the ankles/feet (13.9%) was statistically significantly higher among dental health workers who worked >8 hours compared to dental health workers who worked ≤8 hours (*P* < 0.001, *P* < 0.001, *P* < 0.001, *P* < 0.001, *P* < 0.001, *P* < 0.001, *P* < 0.001, and *P* < 0.001, resp.). Thus, >8 hours worked per day appears to be significantly associated with a higher occurrence of symptoms as shown in Table 3.

The incidence of symptoms in the shoulders (86.4%), the neck (67.8%), the lower back (47.0%), and the hands/wrists (7.58%) was statistically significantly higher among dental health workers who worked >6 days per week compared to dental health workers who worked ≤6 days (*P* < 0.001, *P* < 0.001, *P* < 0.001, and *P* < 0.001, resp.). Thus, the days worked per week of >6 days were significantly associated with a higher incidence of symptoms in the shoulders, the neck, the lower back, the knees, and the ankles/feet symptoms. The occurrence of symptoms in the shoulders (72.3%), the neck (69.2%), the lower back (43.0%), the knees (43.1%), the upper back (32.3%), and the ankles/feet (7.7%) was statistically significantly higher among dental health workers who did no exercise compared to dental health workers who did exercise (*P* < 0.001, *P* < 0.001, *P* < 0.001, *P* < 0.001, *P* < 0.001, and *P* < 0.001, resp.). Thus, the prophylaxis of exercise shows significant association with a reduction of symptoms as is shown in Table 4.

## 4. Discussion

The objective of this research was to evaluate musculoskeletal symptoms among dental health workers. Pain in neck, lower back, shoulders, hands/wrists, hips/thighs, and knees was shown in this study that was significantly more prevalent among dental health workers than other nondental health workers. This finding was consistent with Åkesson et al., 2000 [11], who reported that female dental hygienists and female dentists showed higher prevalence of neck, shoulders, and hands/wrists, compared with their referents during the past 1 year. In addition, this finding was consistent with many studies [8], which compared the prevalence of such symptoms to subjects working in a different environment, such as farmers, pharmacists, and office employees. These symptoms presented more often among dentists. Shoulder complaints were more prevalent than other bodily locations. The symptoms are shown for 3 selected musculoskeletal areas: shoulders, neck, and lower back. The findings for shoulder complaints, neck pain, and lower back pain in this study were supported by Finsen et al. [8], who studied by using a questionnaire that shown to musculoskeletal symptoms among dentists, and variation in dental work. 65% and 59% of dentists replied to neck/shoulder and low back, respectively.

B. Valachi and K. Valachi, 2003 [6], reported that approximately 80% of dentists in America complained of neck, shoulder, and lower back pain. After this, lower back pain was the 2nd most prevalent musculoskeletal area of complaint as presented by many studies [12–14]: 46% reported in a Greek research [15] and 53.7% reported in an Australian research [16]. More than 25% of all subjects reported back pain; it was as severe chronic back pain [12]. In addition, hands/wrist symptoms were significantly more prevalent among dental health workers than other nondental health workers. This finding was consistent with many studies [12, 17–19], which reported hand/wrist complaints among dentists and especially dental hygienists as high. Hand/wrist complaints follow low back disorders [12, 18] and result in a significantly higher chronic situation than any other complaint [12]. Gender was statistically significantly associated with the occurrence of neck, lower back, shoulder, and knee pain (*P* < 0.001, *P* < 0.001, *P* = 0.023, and *P* < 0.001, resp.). The findings for neck, lower back, and shoulder pain in this study was similar to Alexopoulos et al., 2004 [12], who reported that gender was a significant factor for neck pain and that the female gender was more significantly related to chronic back and shoulder pain than male. In addition, all complaints of chronic pain increased with age. In this study, the occurrence of the MSDs was statistically significantly higher among male dental health workers than female dental health workers. This finding was not consistent with many studies [20–22], which reported that the results showed consistency with previous studies that female dental students showed a higher prevalence of WMSD symptoms than males.

Time working included duration of work being hours worked per day and days worked per week that showed a statistically significant correlation with the occurrence of musculoskeletal symptoms. This finding was also supported by Szymanska [17], who reported on illness of the musculoskeletal system among dentists in Poland. This study showed that a long working time in the course of a day was significantly associated with ergonomics. In addition, years of work consequently increase the number of illnesses of the musculoskeletal system, while years of work or experience working time has been significantly associated with increasing disorders of the musculoskeletal system. It is generally accepted that poor practice such as poor posture when working in a long time can be shown to cause chronic fatigue, discomfort, and pain, even if the soft tissues are not structurally altered.

This finding was also supported by Al Wazzan et al. [23], who reported that the disorders of MSDs related with the number of years of dental practice, although the same symptoms were reported in both younger and older dentists. In addition, experiencing pain disorder was significantly earlier with the dentists working in a standing position. This research showed that MSDs occurred within 3 years after working for approximately 10 years of practice and working over 8 hours a day.

Exercise showed a statistically significant reduction in the occurrence of musculoskeletal symptoms. This finding was also supported by Lehto et al. [24], who suggested that most of the dentists did not find as much time as they would like for exercise, a key strategy for reducing dentists' musculoskeletal symptoms. This finding was also supported by Newell and Kumar [25], who reported that using proper body posture, incorporating regular rest breaks, maintaining good general health, performing exercises, and positioning during work can reduce the risk of disorders of MSDs.

The study is based on the self-reporting by dentists. However, the study allowed for a general evaluation of the musculoskeletal symptoms among dentists. In this study, the sample size was limited, with only 124 subjects and 124 references being recruited from 17 community hospitals in Nakhon Si Thammarat province in South of Thailand. Therefore, the results in this study may not be readily comparable beyond this sample group in Thailand.

## 5. Conclusion

In conclusion, musculoskeletal problems among dentists appear to increase with the amount of work they do, and targeted physical exercise appears to counter these problems to some degree. The prevention and reduction of MSDs among dentists should include improving their education in dental ergonomics and increasing their awareness regarding the importance of work related risk factors.

## Figures and Tables

**Table 1:** Occurrence (percent) of musculoskeletal symptoms that interfered with daily activities experienced by dental health workers in the previous 1 year by bodily location and position.

Musculoskeletal symptoms	Count (%) (*n* = 248)	Dental health workers (*n* = 124)	Reference group (*n* = 124)	*P* value
*Trunk*				
Neck	58 (23.4)	48 (38.7)	10 (8.1)	<0.001^*∗*^
Upper back	49 (19.8)	26 (21.0)	23 (18.6)	0.115
Lower back	57 (22.9)	39 (31.5)	18 (14.5)	<0.001^*∗*^
*Arms*				
Shoulders	68 (27.4)	59 (47.5)	9 (7.3)	<0.001^*∗*^
Elbows	28 (11.3)	15 (12.1)	13 (10.5)	0.210
Hands/wrists	35 (14.1)	24 (19.4)	11 (8.9)	<0.001^*∗*^
*Lower body*				
Hips/thighs	25 (10.1)	5 (4.0)	20 (16.1)	<0.001^*∗*^
Knees	32 (12.9)	30 (24.2)	2 (1.6)	<0.001^*∗*^
Ankles/feet	19 (7.7)	11 (8.9)	8 (6.5)	0.250

^*∗*^Significantly associated at *P* value of <0.05.

**Table 2:** Prevalence (percent) of musculoskeletal symptoms that interfered with daily activities experienced by dental health workers in the previous 1 year, by bodily location and gender.

Musculoskeletal symptoms	Count (%) (*n* = 124)	Gender (*n*, %)		*P* value
		Male (*n* = 30)	Female (*n* = 94)	
*Trunk*				
Neck	48 (38.7)	25 (83.3)	23 (24.5)	<0.001^*∗*^
Upper back	26 (21.0)	5 (16.7)	21 (22.3)	0.102
Lower back	39 (31.5)	15 (50.0)	24 (25.5)	<0.001^*∗*^
*Arms*				
Shoulders	59 (47.5)	27 (90.0)	32 (34.1)	<0.001^*∗*^
Elbows	15 (12.1)	2 (6.7)	13 (13.8)	
Hands/wrists	24 (19.4)	5 (16.7)	19 (20.2)	0.115
*Lower body*				
Hips/thighs	5 (4.0)	2 (6.7)	3 (3.2)	0.059
Knees	30 (24.2)	5 (16.7)	25 (26.7)	0.023^*∗*^
Ankles/feet	11 (8.9)	3 (10.0)	8 (8.5)	0.254

^*∗*^Significantly associated at *P* value of <0.05.

**Table 3:** Occurrence (percent) of musculoskeletal symptoms that interfered with daily activities experienced by dental health workers in the previous 1 year by bodily location and duration of work (yrs).

Musculoskeletal symptoms	Count (%) (*n* = 124)	Duration of work (yrs) (*n*, %)		*P* value	Hours worked per day (*n*, %)		*P* value
		≤5 (*n* = 10)	>5 (*n* = 114)		≤8 (*n* = 45)	>8 (*n* = 79)	
*Trunk*							
Neck	48 (38.7)	1 (10.0)	47 (41.2)	<0.001^*∗*^	1 (2.2)	47 (59.5)	<0.001^*∗*^
Upper back	26 (21.0)	2 (20.0)	24 (21.1)	0.257	2 (4.5)	24 (30.4)	<0.001^*∗*^
Lower back	39 (31.5)	1 (10.0)	38 (33.3)	<0.001^*∗*^	5 (11.1)	34 (43.0)	<0.001^*∗*^
*Arms*							
Shoulders	59 (47.5)	2 (20.0)	57 (50.0)	<0.001^*∗*^	6 (13.3)	53 (67.1)	<0.001^*∗*^
Elbows	15 (12.1)	2 (20.0)	13 (11.4)	0.057	0	15 (19.0)	<0.001^*∗*^
Hands/wrists	24 (19.4)	2 (20.0)	12 (10.5)	0.051	2 (4.5)	22 (27.9)	<0.001^*∗*^
*Lower body*							
Hips/thighs	5 (4.0)	0	5 (4.4)	0.551	0	5 (6.3)	0.053
Knees	30 (24.2)	1 (10.0)	29 (25.4)	<0.001^*∗*^	2 (4.5)	28 (34.5)	<0.001^*∗*^
Ankles/feet	11 (8.9)	0	11 (9.7)	<0.001^*∗*^	0	11 (13.9)	<0.001^*∗*^

^*∗*^Significantly associated at *P* value of <0.05.

**Table 4:** Incidence (percent) of musculoskeletal symptoms that interfered with daily activities experienced by dental health workers in the previous 1 months by bodily location and days worked per week.

Musculoskeletal symptoms	Count (%) (*n* = 124)	Days worked per week		*P* value	Exercise (*n* = 59)	No exercise (*n* = 65)	*P* value
		≤6 (*n* = 58)	>6 (*n* = 66)				
*Trunk*							
Neck	48 (38.7)	2 (3.5)	46 (67.8)	<0.001^*∗*^	3 (5.1)	45 (69.2)	<0.001^*∗*^
Upper back	26 (21.0)	12 (20.7)	14 (21.2)	0.524	5 (8.4)	21 (32.3)	<0.001^*∗*^
Lower back	39 (31.5)	8 (13.8)	31 (47.0)	<0.001^*∗*^	17 (28.8)	22 (33.9)	0.057
*Arms*							
Shoulders	59 (47.5)	2 (3.5)	57 (86.4)	<0.001^*∗*^	12 (20.3)	47 (72.3)	<0.001^*∗*^
Elbows	15 (12.1)	7 (12.1)	8 (12.1)	0.624	7 (11.9)	8 (12.3)	0.078
Hands/wrists	24 (19.4)	11 (19.0)	13 (19.7)	0.824	12 (20.3)	12 (18.5)	0.056
*Lower body*							
Hips/thighs	5 (4.0)	0	5 (7.58)	<0.001^*∗*^	0	5 (7.7)	<0.001^*∗*^
Knees	30 (24.2)	11 (19.0)	19 (28.8)	0.042	2 (3.4)	28 (43.1)	<0.001^*∗*^
Ankles/feet	11 (8.9)	5 (8.6)	6 (9.1)	0.512	0	11 (16.9)	<0.001^*∗*^

^*∗*^Significantly associated at *P* value of <0.05.
